# Levonorgestrel-releasing intrauterine device plus metformin, or megestrol acetate plus metformin for fertility-sparing treatment of atypical endometrial hyperplasia and early endometrial carcinoma: a prospective, randomized, blind-endpoint design trial protocol

**DOI:** 10.1186/s12978-022-01513-8

**Published:** 2022-11-04

**Authors:** Xin Zhao, Jumin Niu, Cong Shi, Zhihui Liu

**Affiliations:** Shenyang Women’s and Children’s Hospital, No. 87, Renao Road, Danan Street, Shenhe District, Shenyang, Liaoning China

## Abstract

**Background:**

Endometrial adenocarcinoma (EC) is the fifth most common cancer in women worldwide, standard treatment for EC includes hysterectomy, but it results in the loss of reproductive function. Thus, conservative treatment for these patients is strongly demanded, progestin therapy is widely accepted as the main fertility-sparing treatment for young women with endometrial hyperplasia with atypia (EHA) and well-differentiated endometrioid endometrial cancer. This trial will investigate the effectiveness of conservative treatment for obese women with early-stage EC.

**Method and design:**

This will be an open-label, 2-armed, randomized, phase-II single-center trial of LNG-IUD plus metformin or megestrol acetate (MA) plus metformin. A total of 88 participants will be randomly assigned into 2 treatment arms in a 1:1 ratio. Clinical, laboratory, ultrasound and radiology data, will be collected at baseline, and then at 3, 6, 9, 12, 18, and 24 months. EC biomarkers will be collected at baseline. The primary aim is to determine the efficacy of a levonorgestrel-releasing intrauterine device (LNG-IUD) plus metformin, or megestrol acetate (MA) plus metformin in achieving pathological complete response (pCR) at 12 months, as well as post-treatment pregnancy outcomes and recurrence rate. The secondary aims are to predict the response to an LNG-IUD plus metformin and MA plus metformin via clinical, blood, and tissue predictive biomarkers.

**Conclusions:**

Prospective evidence for conservative treatment of EC is limited. New methods to achieve better CR rates with fewer side effects are needed. This trial will investigate the effectiveness of LNG-IUD plus metformin, and MA plus metformin, in obese women with early-stage EC, providing a non-surgical treatment option for these patients.

*Trial registration* ChiCTR2200055624. The trial was registered at http://www.chictr.org.cn/listbycreater.aspx on January 15, 2022

## Plain Language Summary

Endometrial adenocarcinoma (EC) is the fifth most common cancer in women worldwide, and up to 90% of EC patients are obese. Standard treatment for EC includes hysterectomy, but it leads to loss of reproductive function. This trial will investigate the effectiveness of non-surgical treatment for obese women with early-stage EC. There is limited prospective evidence for the conservative treatment of EC. New methods to achieve better CR rates with fewer side effects are needed. In this trial, patients with early-stage EC will be randomly assigned in a 1:1 ratio to receive treatment with a levonorgestrel-releasing intrauterine device (LNG-IUD) plus metformin, or megestrol acetate (MA) plus metformin. The primary aims are to determine the effectiveness of the 2 treatments to achieve a pathological complete response (pCR) at 12 months, pregnancy outcomes, and recurrence rate. The secondary aims are to predict the response to the 2 treatments using clinical data and blood and tissue predictive biomarkers. If the trial results indicate the treatments are effective, they may significantly reduce the incidence of adverse events in obese EC patients receiving current treatments, and preserve fertility; thereby improving patients’ quality of life and reducing healthcare costs.

## Introduction

Endometrial adenocarcinoma (EC) is the fifth most common cancer in women worldwide [1]. Type 1 EC is typically associated with low-grade tumors with no or infiltrate the superficial muscularis, and with a good prognosis [2]. Endometrial hyperplasia with atypia (EHA) is a pre-malignant lesion of the endometrium, and is a precursor of endometrial cancer. Approximately 30% of women with complex atypical hyperplasia progress to endometrial cancer, and up to 40% of women diagnosed with complex atypical hyperplasia are found to have occult endometrial cancer at the time of hysterectomy [3, 4]. The current standard treatment for EC and EHA is total hysterectomy and bilateral salpingo-oophorectomy, with or without pelvic and para-aortic lymph node dissection [5]. For stage 1 EC, the 5-year disease-free survival (DFS) rate ranges from 80 to 95% [6]. Currently, many women delay childbearing and thus many premenopausal women without children are diagnosed with EC. However, surgical treatment can lead to loss of childbearing ability in women of childbearing age [7–18]. As such, effective non-surgical treatment is beneficial for this population.

Progestin therapy is widely regarded as the main fertility-sparing treatment for young women with EHA and early EC. It involves the use of the oral progestins medroxyprogesterone acetate (MPA) or megestrol acetate (MA), and more recently the placement of a levonorgestrel-releasing intrauterine device (LNG-IUD). Current recommendations are MPA at a dose of 400–600 mg/day or MA at a dose of 160–320 mg/day, with follow-up assessment of treatment response using dilation and curettage (D&C) and imaging studies. The LNG-IUD releases 52 mg of intrauterine progestin at a consistent rate for up to 5 years. MA is associated with higher remission rates compared to MPA and other hormonal treatments, possibly due to the relatively higher bioavailability of MA following oral administration compared to MPA [19]. Placing an LNG-IUD avoids patient noncompliance issues with oral medications and possible side effects associated with high-dose oral progestins [20].

Obesity, diabetes, and insulin resistance are considered risk factors for endometrial cancer. Obese patients also have a 2.5-fold increased risk of death from the disease as compared with non-obese patients [21]. High-dose progesterone is used to treat EHA and early-stage EC in women wishing to maintain fertility. However, high-dose progesterone treatment can lead to further weight gain, increased risk of thrombosis, and impaired liver function. High-dose progesterone is contraindicated if initial testing indicates abnormal liver function [22].

A study has shown that the addition of metformin to progesterone treatment improves the therapeutic effect of progesterone. A recent meta-analysis by Chae-Kim et al. including 6 studies and 621 women demonstrated that for reproductive-aged women with EHA or early-stage EC, the combination of a progestin and metformin is associated with a lower relapse rate, and similar remission, clinical pregnancy, and live birth rates as compared with progestin-alone treatment [23].

Metformin, an insulin sensitizer, is widely used in the treatment of type 2 diabetes and can reduce body weight and activate the AMPK pathway. Activation of the AMPK pathway affects metabolic functions and also inhibits the development of tumors [24, 25]. Clinical studies have shown that metformin exhibits antitumor effects [26, 27]. Schuler et al. conducted a window of opportunity study in which 20 EC women were treated with 850 mg metformin once daily for 1–4 weeks before hysterectomy. The results showed that the mean Ki-67 index (p < 0.008) was significantly decreased pre- to post-treatment [28]. Another study has shown that the expressions of estrogen receptors (ER) and progesterone receptors (PR) are significantly and positively correlated with the expressions of ERK1 and ERK2 in endometrial cancer [29]. These findings suggest that ERs and PRs may participate in the occurrence of EC via the ERK signaling pathway.

We hypothesized that metformin treatment can lead to prolonged PRs, even in the presence of progesterone, which would result in a more durable response to progesterone. In 2021, the feMMe trial (an open-label, randomized phase II clinical trial) demonstrated that the complete pathological endometrial remission rate was 61% for women treated with an LNG-IUD, 57% for women treated with an LNG-IUD plus metformin, and 67% for women treated with an LNG-IUD plus weight loss [30].

However, the outcomes of treatment with an LNG-IUD plus metformin, or MA plus metformin have not been compared. The primary aim of this phase II, 2-arm randomized trial is to determine the effectiveness of an LNG-IUD (Mirena®) plus metformin, and MA plus metformin to achieve a pathological complete response (pCR) at 12th months, pregnancy outcomes, and recurrence rates. The secondary aim is to predict the response to treatment with an LNG-IUD plus metformin, and MA plus metformin using clinical data and blood and tissue predictive biomarkers.

## Patients and methods

### Design

**Fig. 1:**
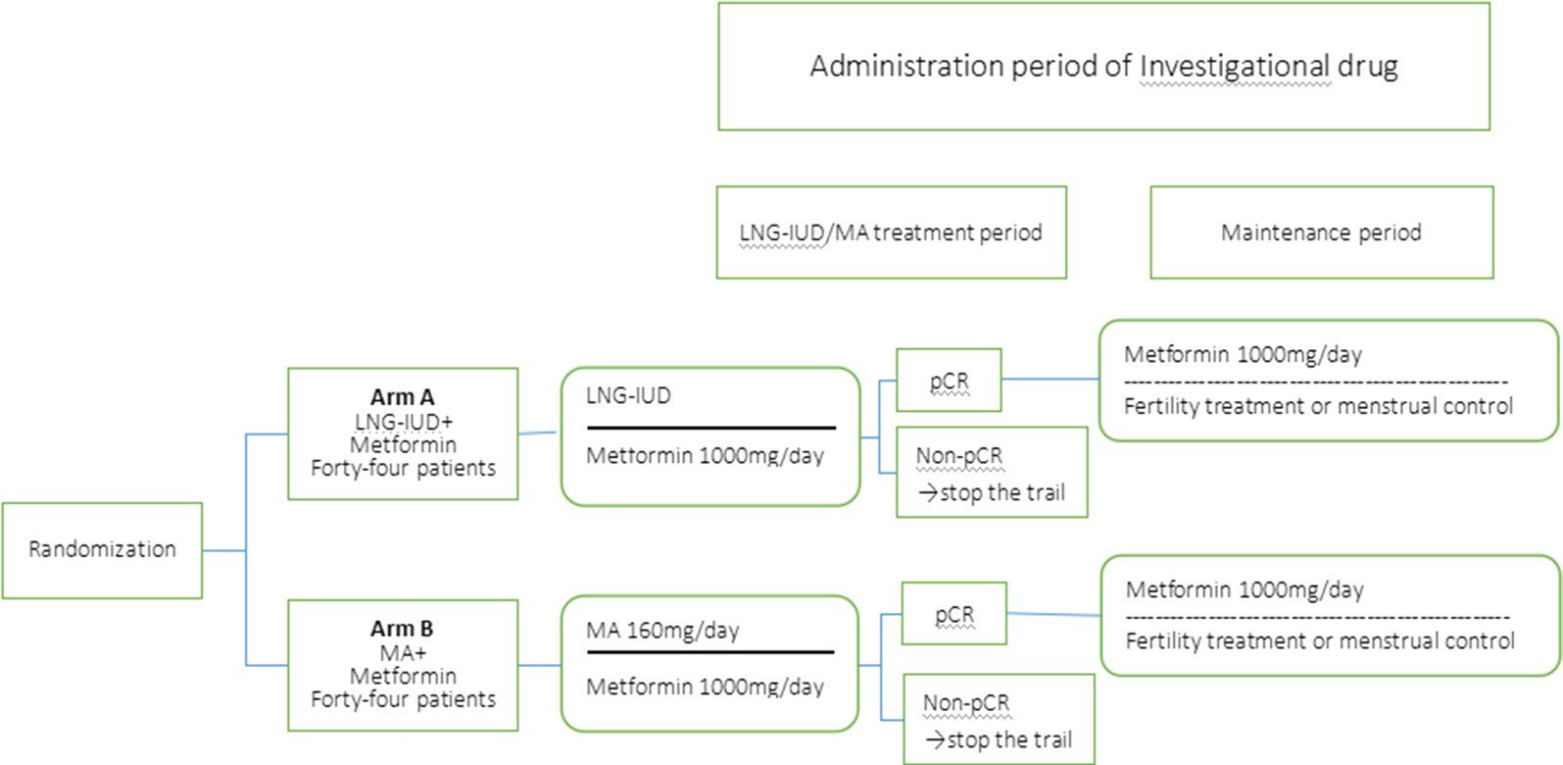
Trial design. pCR, pathological complete response

This trial is designed as an open-label, randomized phase II trial (Fig. 1). The study protocol was approved and registered by the Ethics Committee of Shenyang Women's and Children’s Hospital. The purpose of randomization is to eliminate selection bias.

The trial will only include women with early-stage EC. The criteria for early-stage EC are: (1) stage 1 disease (absence of lymphadenopathy or pelvic masses); (2) endometrioid cell type and International Federation of Obstetrics and Gynecology (FIGO) grade 1 disease; (3) no evidence of lymph vascular space invasion on examination of curettage specimens; (4) absent or only minimal myometrial invasion (depth of infiltration < 1/2 muscle thickness on magnetic resonance imaging [MRI] to exclude deeply invasive cancers); (5) serum cancer antigen (CA)125 level ≤ 30 U/mL [31].

Through strict patient inclusion criteria, we expect to control the probability of disease progression in trial patients at 3%. All patients will receive examinations and laboratory and imaging studies at baseline and 3, 6, 9, 12, 18, and 24 months after starting treatment.

### Patients

Patients who meet the eligibility criteria below will be recruited from our clinic. Oral and written information about the study will be provided by the examining doctor.

### Eligibility criteria

#### Inclusion criteria

- Females with a body mass index (BMI) > 24 kg/m^2^ who wish to maintain fertility.
- Aged over 18 years and under 45 years at the time of randomization.
- No contraindications to pregnancy.
- Histologically confirmed complex EHA or grade 1 EC on a curette or endometrial biopsy tissue specimen.
- Computed tomography (CT) of the pelvis, abdomen, and chest (or chest x-ray) indicates the absence of extrauterine disease.
- MRI shows less than 50% of myometrial invasion (women with EC only).
- No evidence of lymph vascular invasion on curetting specimens.
- FIGO stage IA EC.
- Serum CA 125 level ≤ 30 U/mL.
- No hypersensitivity or contraindications to LNG-IUD, MA, or metformin.
- Ability to comply with endometrial biopsies at specified intervals.
- No use of metformin for at least 2 years before study participation.
- No history of LNG-IUD use.
- No prior use of MAP.
- The primary physician recommended treatment with LNG-IU or MA for EHA or grade 1 EC.
- A negative pregnancy test within 7 days of starting treatment.
- Understanding the study design, risks and benefits, and providing informed consent.

#### Exclusion criteria

- Patients who meet any of the exclusion criteria at baseline will be excluded.
- Eastern Cooperative Oncology Group (ECOG) performance status > 3.
- MRI shows grade 1 EC with myometrial invasion > 50%, grade 2 or grade 3 EC.
- Histological cell type other than EC (sarcoma or high-risk cell type, e.g., papillary serous or clear cell).
- Prior treatment for EC or EHA.
- A history of pelvic or abdominal radiotherapy.
- Renal impairment (creatinine > 150 µmol/L [1.7 mg/dL]), acute pulmonary edema, or liver failure.
- Unwilling to have additional endometrial biopsies, or unable to attend 3 monthly clinical assessments.
- Unable to provide informed consent or complete questionnaires.
- Medical imaging shows evidence of extrauterine spread.
- A congenital or acquired uterine anomaly which distorts the uterine cavity.
- Acute pelvic inflammatory disease.
- Concurrent with different cancer.
- Chronic (daily use for > 1 month) use of cimetidine (significant increase in metformin concentration and risk of lactic acidosis).
- Use of Iodinated contrast agents within the past 48 h (significant increase in metformin concentration and risk of lactic acidosis).
- Pregnant or lactating.
- History of hypersensitivity to metformin or discontinuation due to adverse effects.
- High risk of thrombosis.
- Surgery within a week before starting treatment.
- Current or history of cerebral and/or cardiac infarction or thrombophlebitis.
- Valvular heart disease, atrial fibrillation, endocarditis, or other serious cardiac conditions.
- Currently receiving hormone treatments.

### Study assessments and procedures

The study assessments and procedures performed at baseline and 3, 6, 9, 12, 18, and 24 months are summarized in Table 1.

**Table 1:** Schedule of patient assessments

	Baseline	Day 1	3 months (± 14 days)	6 months (± 14 days)	9 months (± 14 days)	12 months (± 14 days)	18 months (± 14 days)	24 months (± 14 days)
Assessment								
Informed consent	√							
CT of pelvis/abdomen^a^	√			√		√	√	√
CT chest (or chest X-ray)	√			√		√	√	√
MRI of pelvis	√			√		√	√	√
Pelvic ultrasonography^b^	√		√	√	√	√	√	√
CA125	√		√	√	√	√	√	√
FBG (fasting blood-glucose)	√		√	√	√	√	√	√
Serum or urine pregnancy test	√	√(urine)						
Fasting blood sugar	√		√	√	√	√	√	√
HbA1c	√		√	√	√	√	√	√
HE4 (human epididymal protein4)	√		√	√	√	√	√	√
Blood collection for biomarkers and genetic testing	√							
ERα, PRα and PRβ (estrogen receptor, progesterone receptor)								
Medical history	√							
Concomitant medications	√		√	√	√	√	√	√
ECOG	√		√	√	√	√	√	√
Height	√							
Weight	√		√	√	√	√	√	√
BMI	√		√	√	√	√	√	√
Surgical, medical, gynecologic, and family history	√							
Self-efficacy and social support questionnaire	√		√	√	√	√	√	√
Adverse events			√	√	√	√	√	√
Hysteroscopy, D&C			√	√	√	√	√	√
Intervention adherence			√	√	√	√	√	√

*BMI* body mass index, *CA125* cancer antigen 125, *CT* computed tomography, *D&C* dilation and curettage, *ECOG* Eastern Cooperative Oncology Group, *HbA1c* glycosylated hemoglobin, *MRI* magnetic resonance imaging

^a^CT scans will be performed every 6 months. However, if the ultrasound shows obvious lesions in the uterine cavity and tissue pathological examination indicates no remission or the disease has progressed, additional CT examination will be performed

^b^Patients will receive pelvic ultrasonography and hysteroscopic and curettage every 3 months for endometrial pathological examination. If the ultrasound shows obvious lesions in the uterine cavity and tissue pathological examination indicates no remission or the disease has progressed, additional CT/MRI examination will be performed

### Interventions

The LNG-IUD is approved for contraception, treatment of idiopathic menorrhagia, and prevention of endometrial hyperplasia. The most common adverse reactions are uterine/vaginal bleeding alterations, amenorrhea, intermenstrual bleeding and spotting, abdominal/pelvic pain, and ovarian cysts. The device is prohibited for use in women with known or suspected pregnancy, congenital abnormal uterine development, breast cancer, unexplained vaginal bleeding, reproductive tract infection, and other conditions.

Systemic progestogen therapy, such as MA 160 mg orally once a day, is effective for the treatment of hormone-sensitive hyperplasia and tumors [32]. However, PRs are often downregulated leading to a relatively short duration of effectiveness [33]. In addition, systemic therapy is associated with low compliance rates due to adverse effects including nausea, thromboembolic complications such as deep vein thrombosis (DVT), pulmonary embolism (PE), stroke, weight gain, abnormal vaginal bleeding, and increased risk of breast cancer [34].

Study participants will be instructed to take metformin with meals, 1000 mg daily. This dose is similar to that used in clinical trials as an adjuvant breast cancer treatment (NCT00909506 and NCT01302002), ranging from 500 to 1000 mg daily. Metformin is well-tolerated by the vast majority of patients with no significant side effects. The gastrointestinal adverse effects of metformin are dose-dependent [35]. The dose of 1000 mg daily can be reduced to 500 mg daily if needed. Metformin is associated with abdominal pain, diarrhea, lactic acidosis, nausea and vomiting, and taste changes [35]. Metformin decreases absorption of vitamins B9 and B12, in turn elevating homocysteine levels. Metformin is not used for patients with moderate or worse renal function impairment.

Because all participants in the trial will be obese, a weight loss program will be implemented. The weight loss program is an evidence-based and tested diet and exercise intervention program that has led to weight loss success in obses people [36]. Exercise levels (sedentary, moderately active, and sufficiently active) will be measured using the Active Australia Survey [37].

### Randomization

Patients will be randomly assigned to arm A (LNG-IUD plus metformin) or arm B (MA plus metformin) in a 1:1 ratio by an internet-based distant third-party statistician blinded to the study and participant details. The doctor involved in recruitment will receive training and instructions on the recruitment procedure.

### Treatment of adverse events

Any adverse events will be managed by local investigators according to current good clinical practice guidelines. The details of each adverse event will be described in a case report form, including the nature of the adverse event, time of onset and time of resolution, severity, treatment, and outcome. If necessary, a followed-up examination will be performed to ensure patient safety.

If the physician monitoring the trial finds evidence of harm to a participant or signs of ineffectiveness, the participant will be withdrawn from the study. The results of these participants will be analyzed as a non-pCR group.

### Criteria for discontinuation of trial treatment

The criteria for the discontinuation of a trial medication are as follows:

1. A participant declines further participation or withdraws their consent.
2. Cancelation of the entire study.
3. The protocol treatment will be stopped if it does not result in remission according to the following criteria: no treatment response or pCR by 1 year; disease progression at any time; relapse after remission.
4. Severe adverse events (progressive or persistent) which may be related to the medication (such as hemorrhagic shock due to massive vaginal bleeding, severe allergy, thrombosis, liver function damage) and newly diagnosed other malignancy (e.g., breast cancer) will be evaluated by 2 chief physicians before the trial is stopped.
5. Any situation in which the LNG-IUD, MA, or metformin treatment cannot be continued according to the judgment of the physician.

### Discontinuation of the study

The study will be terminated early if the IRB determines the occurrence of any of the following: serious adverse drug effects (abnormal liver function unresponsive to treatment, thrombus, hemorrhagic shock due to massive bleeding, severe allergic reaction); other newly diagnosed malignancy; the participants with unexpected, significant or unacceptable risks (such as death); the trial treatment is determined to be ineffective.

### Baseline assessments

Baseline assessments will be performed according to the trial standard operating procedure (SOP), including medical history, determination of BMI, CT and MRI imaging, blood tests including tumor marker CA125. Endometrial tissue specimens will be obtained by treating gynecologic oncologists via hysteroscopy and D&C. Histopathological examination of tissue specimens will confirm the cell type and grade of differentiation. Histopathological specimens will also be used for pathological molecular typing.

### Efficacy assessments

Assessments of effectiveness will be performed at 3, 6, 9, 12, 18, and 24 months after beginning treatment, including weight loss, histological examination of endometrial tissue specimens, and pregnancy outcomes. The total study duration will be 2 years.

**Fig. 2:**
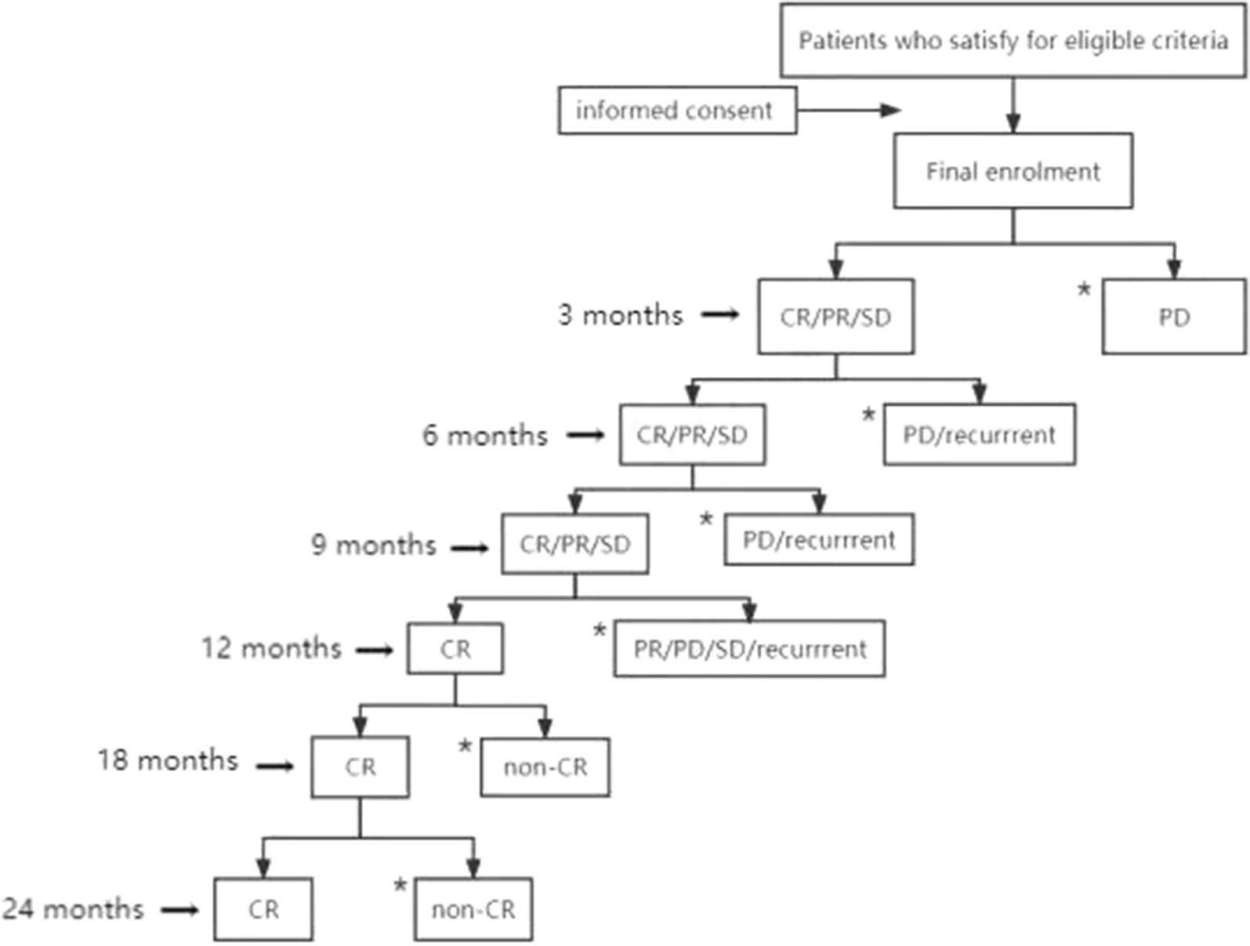
Assessment of pCR during treatment. *CR* complete response, *PR* partial response, *SD* stable disease, *PD* progressive disease. *Protocol treatment is halted

1. Weight loss of patients in the 2 groups will be evaluated.
2. Hysteroscopic endometrial curettage will be performed to rule out disease progression (Fig. 2).
3. Complete pathological response is defined as 2 consecutive negative endometrial biopsies. Endometrial tissue for pathological examination will be obtained during hysteroscopy. Tissue pathological examination will be performed in a double-blind manner by 2 senior pathologists. A third pathologist will be consulted if the results are inconsistent.
  A. If the disease progresses, such as atypical hyperplasia to endometrial cancer, well-differentiated adenocarcinoma of the endometrial to moderately or poorly differentiated endometrial cancer, or other types of endometrial cancer, hysterectomy will be recommended.
  B. If complete remission is achieved (2 consecutive negative endometrial biopsies), the patient will be recommended to remove the LNG-IUD or discontinue oral progesterone. Patients will continue to take metformin and monitor ovulation if pregnancy is desired. Participants who are not pregnant will be followed-up every 3 months for endometrial biopsy. Patients with a successful pregnancy and delivery who desire to retain reproductive function will undergo regular endometrial biopsies and imaging studies.
  C. If there is no response to treatment, but no progression or partial response, the current treatment will be continued; assessments and efficacy evaluations will be performed every 3 months. If there is progression or recurrence after remission, the patient will be recommended to terminate participation in the trial. If the assessments continue to indicate no response or partial response, assessments will be continued every 3 months for 12 months. If assessment at 12 months indicates an incomplete response, the patient will be recommended to terminate participation in the trial.
  D. If EC develops during follow-up after a complete response, it will be considered a recurrence and surgical treatment will be recommended.
  E. If there is no improvement or partial response after 12 months of treatment, the patient will be informed of the risks of continuing conservative treatment and a hysterectomy will be recommended.
4. Pregnancy assessment: outcomes of abortion, premature delivery, and full-term delivery will be recorded.

### Follow-up evaluations

At the 3-month follow-up, if endometrial pathology shows progressive disease, the patient will be withdrawn from the trial. If the endometrial pathology indicates a complete response, partial response, or stable disease, the patient will be followed-up after 3 months. At the 6 month follow-up, if the endometrial pathology shows progressive disease or recurrence, the patient will be withdrawn from the trial. If the pathology shows a complete response, partial response, or stable disease, the patient will be followed-up after 3 months. At the 9-month follow-up, if the endometrial pathology shows progressive disease or recurrence, the patient will be withdrawn from the trial. If the pathology shows a complete response, partial response, or stable disease, the patient will be followed-up after 3 months. At the 12-month follow-up, if the endometrial pathology shows a partial response, progressive disease, stable disease, or recurrence, the patient will be withdrawn from the trial. If the pathology shows a complete response, the patient will be followed-up in 6 months. At the 18-month follow-up, if the endometrial pathology shows an incomplete response, the patient will be withdrawn from the trial. If there is a complete response, the patient will be followed-up after 1 year. At the 24-month follow-up, if the endometrial pathology shows an incomplete response, the patient will be withdrawn from the trial. In the case of a complete response, the patient will continue to be followed long-term.

The study duration is 2 years, but the treatment and follow-up of patients will be long-term. A hysterectomy will be recommended for patients who have a complete response and have completed childbirth. Patients who have fertility requirements or are unwilling to undergo a hysterectomy will be followed closely for signs of recurrence.

### Data monitoring committee

The data monitoring committee (DMC) will be composed of clinical trial specialists, including a biostatistician, who are not associated with the study. The committee will meet at least twice a year, and all data obtained from the trial will be evaluated by the committee.

The DMC will review unblinded outcome data for safety and efficacy, and judge if there is evidence that either treatment is unsafe and the trial should be discontinued. The DMC will also advise the Trial Steering Committee of any evidence of unethical treatment or unacceptably serious adverse events.

The IBM Clinical Development Management System (IBM Corporation, Somers, New York), which is based on an EDC (Electronic Data Capture System), will be used to manage the data in this study.

### Sample size

According to a previous study, the mean pCR rate for LNG-IUD alone was 68% (95% confidence interval [CI]: 45–86%) [30], it was assumed that the rate in the MA-alone group will be 60%. Based on a prior study [38], we anticipate that the true pCR rate will be closer to the lower confidence limit of 45%. Patients who become pregnant during the trial will be withdrawn from the study and treated as non-recurrence in-trial cases.

The sample size was estimated by software G*Power version 3.1 (Heinrich-Heine-Universität Düsseldorf, Düsseldorf, German). The effect size, w, of the 2-group contingency table was set at a medium level of 0.3, type I error (alpha) at 0.05, 2-tailed, and power was set at 0.80, chi-square test. The size ratio between the 2 groups was set at 1:1. The estimated minimum required sample size of each group is 44, and the total sample size is 88.

### Statistical analysis

The EHA and EC pCR rates and 95% CIs will be calculated for both groups. Patient’s quality of life will be assessed at baseline, week 1, and at 3, 6, 9, 12, 18, and 24 months. Other data acquisition and analyses will include the complete response rate for the trial treatment period; the rate of trial continuation for the LNG-IUD plus metformin and MA plus metformin groups; BMI values and homeostatic model assessment of insulin resistance (HOMA-IR) results of all response cases that wish to become pregnant; the proportion of patients that become pregnant at least once during the trial period; pregnancy outcomes (miscarriage, stillbirth, live birth, and weeks of gestation); and the proportion of patients that give birth to a child. Data will be reported as mean and standard deviation (SD), median and interquartile range (IQR), or number and percentage (%) based on if the data are continuous or categorical and on the normality assumption. The Kolmogorov–Smirnov normality test will be used to check the normality of continuous variables. For comparisons between 2 groups, Student’s t-test or the Mann–Whitney U test will be used according to the normality assumption. The Chi-square test will be used to compare the number of cases and percentage between groups.

This study will also investigate changes in the expression of molecular biomarkers. Since the changing results over time are repeated measurements, statistical tests of dependent data, such as the paired t-test or mixed-design ANOVA will be used.

## Discussion

The current trial is innovative in that it will test the efficacy of 2 interventions (LNG-IUD plus metformin, MA plus metformin) in the context of phase II randomized design. Obese patients with low-grade and early-stage EC or EHA will be enrolled because the prognosis of these patients is excellent, and even non-responders will likely be harmed by withholding a definitive treatment for 12 months [30].

A meta-analysis of outcomes after progestin treatment found oral and LNG-IUD treatments were similarly effective [38]. In patients with stage 1 EC treated with intrauterine progestins, the weighted mean pCR rate was 68% (95% CI 45–86%), but this was only based on one published and one unpublished series. Oral progesterone therapy for EC is associated with side effects, including thromboembolic complications (DVT, PE, stroke), weight gain, and the onset or worsening of type 2 diabetes mellitus [38]. In contrast, the LNG-IUD is not known to cause systemic side effects; however, recurrence is common after LNG-IUD removal [39, 40].

The current study is also innovative because it will test combinations of drugs to metabolic imbalances in endometrial cancer patients. Metformin is an oral hypoglycemic agent and insulin sensitizer, which can reduce body weight and activate the AMPK pathway [41, 42]. Activation of the AMPK pathway affects metabolic functions and also inhibits the development of tumors [7, 10, 11, 13, 18]. In a study using nude mice and cultured EC cells, mice treated with metformin had significantly reduced tumor volume at autopsy compared with control mice [28]. Schuler et al. reported 20 cases of EC in which metformin was administered at a dosage of 850 mg daily for 1–4 weeks before hysterectomy, and Ki-67 levels were significantly lower postoperatively than preoperatively [28].

Retrospective case series and meta-analyses on the use of LNG-IUD for EHA and EC have been published. In addition, a number of prospective, randomized and non-randomized clinical trials of using an LNG-IUD are ongoing. Trial NCT01594879 (clinical trial.gov) is a single-arm, non-randomized clinical trial examining the efficacy of LNG-IUD and MPA to treat patients with EHA and EC. A total of 39 patients are planned to be enrolled, the study outcome is pathological response at 24 months. The USC/Norris Comprehensive Cancer Center will conduct a phase 2 randomized controlled trial (NCT01943058) enrolling 130 patients to receive either MA or an LNG-IUD for 18 months. In 2014, Trial NCT02035787 was begun, which is an open-label, single-arm, single-center study of adding metformin to an LNG-IUD for the treatment of 30 non-surgical patients with either EHA or grade 1 EC. The outcome is pathological results at 6 months, and the results will be published in 2022.

There are also several ongoing or forthcoming trials of metformin in EC women. For example, Trial NCT04792749 is to investigate the effects of metformin in addition to conventional progestin therapy in the fertility-sparing treatment of early-stage EC. The Gynecologic Oncology Group (GOG) is comparing paclitaxel, carboplatin, and metformin to paclitaxel, carboplatin, and placebo for the treatment of stage 3 or 4 or recurrent EC (NCT02065687). Lu et al. from the MD Anderson Cancer Center are investigating whether metformin and/or a lifestyle intervention can prevent EC in obese post-menopausal women (NCT01697566). In 2013, NCT01968317 was begun comparing metformin plus MA with MA alone as a fertility-sparing treatment in patients with EHA and well-differentiated EC. The results showed that metformin plus MA was associated with a higher early CR rate than MA alone in patients with EHA. Metformin is inexpensive and has an excellent safety profile, so it appears to be an obvious choice for a prospective randomized study. Nevertheless, an appropriate dosage of MA in combination with megestrol and metformin has not been determined. According to current clinical guidelines for fertility-sparing therapy, the dosage of MA is 160–320 mg/day. However, high-dose progesterone therapy can lead to further weight gain, increases the risk of thrombosis, and impairs liver function. In addition, patients with abnormal liver function cannot receive high-dose progesterone. Therefore, we chose a MA dosage of 160 mg/day.

Matsuo et al. reported that for patients with endometrial dysplasia, concurrent metformin administration was associated with an improved response in women using the LNG-IUD as compared with those receiving metformin plus progesterone (oral progesterone/LNG-IUD) treatment [43].

This proposed trial has strengths and weaknesses. A marked strength is that no high-quality studies are comparing the efficacy of an LNG-IUD plus metformin or MA plus metformin in the treatment of early EC. In other relevant studies, the primary endpoint is the pathological response rate while in the proposed trial the endpoints will be the pCR rate, pregnancy outcomes, and recurrence rates. The proposed trial is to investigate whether metformin combined with local progesterone and systemic progesterone have different therapeutic effects on endometrial lesions and whether there are different pathways for their “sensitization” effects. Finally, in ongoing trials (NCT01594879, NCT01943058, NCT02035787, NCT04792749, NCT02065687, NCT01968317, NCT01697566), metformin is administered in combination with progestin, and is discontinued after remission. In these trials, only an anticancer effect of metformin is expected as an outcome. Since most of the participants of this trial will be obese, they are likely to develop insulin resistance and abnormal glucose metabolism. Thus, this trial will also evaluate the expected improvements in the metabolic profile following the addition of metformin. Strengths of our work also include its prospective design, standardized treatment protocol, and long duration of follow-up.

A limitation of this trial is that the design is not that of a confirmatory trial. In addition, this trial will not use a placebo control group. However, evaluation of the remission and relapse rates, which are associated with the primary endpoint, will be performed by a pathological review board. The evaluation will be conducted under blind and independent conditions. Therefore, we believe that it will be possible to maintain objectivity and reduce potential bias.

We hope that the current study will increase the level of evidence on the effectiveness of an LNG-IUD in treating early EC and EHA. We hope that the LNG-IUD may be recognized as an effective alternative therapy for EC and EHA in obese women wishing for fertility preservation.

## Conclusion

This trial will investigate the effectiveness of an LNG-IUD plus metformin, and MA plus metformin, in obese women with early-stage EC, providing a non-surgical treatment option for these patients.

## Data Availability

Requests for data generated during this study will be considered by DMC. Data will typically be available within six months after the primary publication. Only scientifically sound proposals from appropriately qualified Research Groups will be considered for data sharing. The request will be reviewed by the Data Sharing Committee in discussion with the Chief Investigator and, where appropriate (or in absence of the Chief Investigator) any of the following: the Trial Sponsor, the relevant Trial Management Group (TMG), and independent Trial Steering Committee (TSC). A formal Data Sharing Agreement (DSA) may be required between respective organizations once the release of the data is approved and before data can be released. Data will be fully de-identified (anonymized) unless the DSA covers the transfer of patient identifiable information. Any data transfer will use a secure and encrypted method.

## Abbreviations

EC: Endometrial adenocarcinoma
LNG-IUD: Levonorgestrel-releasing intrauterine device
MA: Megestrol acetate
pCR: Pathological complete response
EHA: Endometrial hyperplasia with atypia
SOP: Standard operating procedure
D&C: Dilatation and curettage
DVT: Deep vein thrombosis
PE: Pulmonary embolism
